# Overexpression of platelet-derived growth factor receptor α in breast cancer is associated with tumour progression

**DOI:** 10.1186/bcr1304

**Published:** 2005-08-01

**Authors:** Inês Carvalho, Fernanda Milanezi, Albino Martins, Rui M Reis, Fernando Schmitt

**Affiliations:** 1 IPATIMUP – Institute of Molecular Pathology and Immunology of Porto University, Porto, Portugal; 2 Life and Health Sciences Research Institute (ICVS), School of Health Sciences, University of Minho, Braga, Portugal; 3 Medical Faculty of Porto University, Porto, Portugal

## Abstract

**Introduction:**

Receptor tyrosine kinases have been extensively studied owing to their frequently abnormal activation in the development and progression of human cancers. Platelet-derived growth factor receptors (PDGFRs) are receptors with intrinsic tyrosine kinase activity that regulate several functions in normal cells and are widely expressed in a variety of malignancies. After the demonstration that gastrointestinal stromal tumours without c-Kit mutations harbour PDGFR-α-activating mutations and that PDGFR-α is also a therapeutic target for imatinib mesylate, the interest for this receptor has increased considerably. Because breast cancer is one of the most frequent neoplasias in women worldwide, and only one study has reported PDGFR-α expression in breast carcinomas, the aim of this work was to investigate the potential significance of PDGFR-α expression in invasive mammary carcinomas.

**Methods:**

We used immunohistochemistry to detect PDGFR-α overexpression on a series of 181 formalin-fixed paraffin-embedded invasive ductal breast carcinomas and in two breast cancer cell lines: MCF-7 and HS578T. We associated its expression with known prognostic factors and we also performed polymerase chain reaction–single-stranded conformational polymorphism and direct sequencing to screen for PDGFR-α mutations.

**Results:**

PDGFR-α expression was observed in 39.2% of the breast carcinomas and showed an association with lymph node metastasis (*P *= 0.0079), HER-2 expression (*P *= 0.0265) and Bcl2 expression (*P *= 0.0121). A correlation was also found with the expression of platelet-derived growth factor A (PDGF-A; *P *= 0.0194). The two cell lines tested did not express PDGFR-α. Screening for mutations revealed alterations in the PDGFR-α gene at the following locations: 2500A→G, 2529T→A and 2472C→T in exon 18 and 1701G→A in exon 12. We also found an intronic insertion IVS17-50insA at exon 18 in all sequenced cases. None of these genetic alterations was correlated with PDGFR-α expression. The cell lines did not reveal any alterations in the PDGFR-α gene sequence.

**Conclusion:**

PDGFR-α is expressed in invasive breast carcinomas and is associated with biological aggressiveness. The genetic alterations described were not correlated with protein expression, but other mechanisms such as gene amplification or constitutive activation of a signalling pathway inducing this receptor could still sustain PDGFR-α as a potential therapeutic target.

## Introduction

Uncontrolled tumour cell proliferation due to abnormal activation of several growth factors and their receptors is relevant in the events underlying human cancer development, because the tyrosine kinases receptors form one of the most important classes of growth factor receptors implicated in that process. Platelet-derived growth factor receptors (PDGFRs) α and β are characterized by an intracellular tyrosine kinase domain whose activation depends on ligand binding. The platelet-derived growth factor (PDGF) family of growth factors consists of five different disulphide-linked dimers, PDGF-AA, -BB, -AB, -CC and -DD that act via the two receptors PDGFR-α and PDGFR-β. All PDGF isoforms except PDGF-DD induce PDGFR-α dimerization, although this receptor binds to PDGF-AA with higher affinity, whereas PDGF-BB and PDGF-DD activate PDGFR-β dimers. After receptor activation, several intracellular pathways are stimulated, leading to cell proliferation and several other crucial processes [1]. PDGFR signalling has important functions during embryogenesis, and its overexpression is associated with several pathological conditions such as fibrotic and vasculoproliferative diseases and cancer [2-5]. Recently, the finding that gastrointestinal stromal tumours (GISTs) lacking c-Kit mutations harbour intragenic activating mutations in PDGFR-α [6,7], and that ligand-independent constitutive activation can be blocked by means of a tyrosine kinase inhibitor (imatinib mesylate), has increased the interest for PDGFR-α as a target for therapy.

The gene encoding PDGFR-α is located at chromosome 4q11-12, which spans 23 exons and encodes a transmembrane protein composed of five immunoglobulin-like domains in the extracellular region, a transmembrane domain, an ATP binding site and a hydrophilic kinase insert domain in the intracellular portion [8].

Despite increased public awareness, screening programmes and early detection, breast cancer remains the second leading cause of cancer death in women. This leads to a constant search for new biological markers that could be used as prognostic/predictive factors and therapeutic targets, resulting in better disease-free survival and overall survival [9]. Given the success of imatinib mesylate therapy of chronic myeloid leukaemia and GIST, the molecular targets for this drug have been explored in distinct types of cancer.

To the best of our knowledge, there have been few studies on PDGFRs in mammary neoplasias and no reports on the presence of PDGFR-α mutations in breast carcinomas. The aims of the present study were the following: first, to evaluate the immunohistochemistry expression of PDGFR-α and PDGF-A in a series of invasive ductal breast carcinomas; second, to correlate the PDGFR-α expression with prognostic factors in breast cancer; and third, to screen for PDGFR-α gene-activating mutations in breast cancer.

## Materials and methods

### Tissue specimens

One hundred and eighty-one formalin-fixed paraffin-embedded cases of invasive ductal breast carcinomas were retrieved from the histopathology files of IPATIMUP and São João Hospital (Porto, Portugal). All cases were independently reviewed on haematoxylin/eosin-stained sections by two pathologists (FS and FM). All relevant data were available for analysis, including age, tumour size, histological grade, axillary lymph node status, oestrogen receptor status, p53, MIB-1 and HER-2 expression, angiogenic index and patient survival.

The mean age of the patients was 55 years old (range 24 to 83) and the size of the tumours ranged from 2.0 to 150.0 mm (mean 30.9).

### Cell lines

MCF-7 and HS578T breast cancer cell lines (ATCC, Teddington, UK) were maintained in Eagle's minimum essential medium and Dulbecco's modified Eagle's medium, respectively, and were supplemented with 10% heat-inactivated fetal bovine serum, 100 U/ml penicillin and streptomycin (Gibco, Paisley, UK) in a humidified incubator at 37°C with a 5% CO_2 _atmosphere.

The medium was replaced every 2 to 3 days in all cell cultures.

Cell lines were grown until confluence; they were then scraped and the suspension was transferred into a tube for cell block preparation. After centrifugation at 1,800 r.p.m. for 5 min, the supernatants were carefully removed without dislodging the cell button. After the addition of 10 ml of 10% neutral buffered formalin to the intact cell button, the mixture was kept at room temperature (18 to 25°C) for 20 min. An additional centrifugation at 1,800 rpm for 5 min was followed by removal of the formalin supernatant and the addition of two or three drops of bovine albumin (22% from Ortho Diagnostics). After mixing, 10 ml of 95% ethanol was added to the sample, which was mixed again. After a final centrifugation step, the mixture rested for 15 min. The button was carefully loosened to allow it to be slipped intact out of the tube, and the solution was poured through lens paper to filter it. The cell button was wrapped in tissue paper and placed in a tissue cassette, which was kept in 10% neutral buffered formalin until further processing and paraffin embedding.

### Immunohistochemistry

Automated immunohistochemistry (Lab Vision Autostainer LV-1; Lab Vision Corporation, Fremont, CA, USA) was performed with the streptavidin–biotin–peroxidase technique, using antibodies raised against human PDGFR-α (1:200 dilution; Lab Vision Corporation) and PDGF-A (clone N-30; 1:80 dilution; Santa Cruz Biotechnology, Santa Cruz, CA, USA). In brief, antigen retrieval was performed in 10 mM citrate buffer (pH 6.0) for 20 min with wet heat (hot bath) at 98°C for PDGFR-α or, for PDGF-A, samples were pretreated in 10 mM citrate buffer for 15 min (3 x 5 min) in a microwave at 600 W. After cooling to room temperature, the sections were rinsed with PBS, which was used for all subsequent washing steps. Endogenous peroxidase activity was blocked by the incubation of slides in 3% hydrogen peroxide in methanol for 10 min, and non-specific epitopes were eliminated by incubation with a blocking solution (UltraVision Block; Lab Vision Corporation) for 10 min. The slides were incubated with the primary antibodies for 30 min. After the slides had been rinsed, they were incubated with biotinylated secondary antibody followed by enzyme-labelled streptavidin for 10 min (UltraVision detection system, anti-polyvalent horseradish peroxidase/diaminobenzidine; Lab Vision Corporation). The immunoreaction was developed with diaminobenzidine (LabVision Corporation). Slides were counterstained with Gill's haematoxylin.

A positive control was included in each slide run. Sections of vessels in the corion (lamina propria) of gastrointestinal mucosa biopsies were used as positive controls, and blood vessels present in the periphery of the carcinomas studied were used as internal positive controls. The cases were considered positive whenever there was cytoplasmic staining for PDGFR-α and PDGF-A.

### Statistical analysis

Invasive ductal breast carcinomas were subclassified into cases with or without PDGFR-α and PDGF-A expression. Contingency tables and the χ^2 ^test were used in StatView 5.0 software (SAS Institute Inc., Cary, NC, USA) to estimate the correlation between PDGFR-α immunoreactivity and clinical-pathological and molecular markers previously studied for these cases. A correlation was considered significant whenever *P *< 0.05.

### Mutation analysis

Screening for mutations by polymerase chain reaction–single-stranded conformational polymorphism (PCR-SSCP) followed by direct DNA sequencing was performed in only 13 cases from our series (because of the unavailability of biological material necessary for this study), and in the two cell lines.

#### DNA extraction

The tumour tissue was microdissected with a sterile scalpel under a stereomicroscope to avoid contamination with non-neoplastic tissues. DNA extraction was performed with the NucleoSpin kit (Macherey-Nagel, Düren, Germany) for blocks embedded in paraffin wax.

For the human cell lines MCF-7 and HS578T, DNA was extracted with the salting-out procedure, with some modifications. In brief, cell pellets were dissolved in SE buffer (1.2 M sorbitol, 0.1 M EDTA, pH 8.0) and digested overnight with proteinase K. After incubation, saturated NaCl and chloroform were added to allow the separation of proteins from DNA. DNA precipitation was performed with propan-2-ol. Finally, DNA was washed with 70% ethanol and dissolved in TE buffer (10 mM Tris/Cl, 1 mM EDTA, pH 8.0). The concentrations were determined by spectrophotometry and aliquoted DNA was stored at -20°C until use.

#### PCR-SSCP

Pre-screening of exons 12 and 18 of the PDGFR-α gene was performed with the primers with sequences shown in Table 1 and described elsewhere [6]. PCR was performed with 2 to 4 μl of DNA solution, PCR buffer (16 mM (NH_4_)_2_SO_4_, 67 mM Tris-HCl pH 8.8, 0.01% Tween-20; Bioron GmbH, Ludwigshafen, Germany), 1.5 to 2.5 mM of MgCl_2 _(Bioron GmbH), each dNTP (Fermentas, Ontario, Canada) at 0.2 mM, each primer at 0.2 μM, and 1 U/μl SuperHot Taq DNA polymerase (Bioron GmbH) in a final volume of 25 μl. Thirty-seven cycles of denaturation (95°C), annealing (58 to 60°C) and extension (72°C), for 45 s each, were performed in a gradient thermocycler (Bio-Rad, Hercules, CA, USA).

A total of 20 μl of the PCR products was mixed with an equivalent volume of denaturing loading buffer (98% formamide, 0.05% xylenecyanol and bromophenol blue). The samples were denatured at 95°C for 10 min, put into ice and run at 4°C in a 0.8 × MDE gel for exon 12 and in a 0.8 × MDE gel with 3% glycerol for exon 18 at 200 to 280 V, for 16 to 20 hours.

Gels were fixed in 10% ethanol for 10 min and oxidized in 1% nitric acid solution. After 3 min, gels were stained in the dark with a 0.012 M silver nitrate for 20 min. The gels showed an appropriate colour in 0.28 M anydrous sodium carbonate and 0.019% formalin. The reaction was stopped by incubation in 10% acetic acid for 2 min. Subsequently, gels were dried at 80°C for 2 hours (Thermo Savant SGD5040 Slab Gel Dryer).

#### Direct sequencing

The abnormal PCR products for each exon screened by SSCP were purified (MicrospinTM S-400 HR columns; Amersham Biosciences, Little Chalfont, Bucks., UK) and analysed by direct sequencing (Sequence Laboratories Göttingen GmbH, Göttingen, Germany). For cell lines, after being purified, the PCR products were subjected to a cycle sequencing reaction with an ABI PRISM BigDye Terminator Cycle Sequencing Ready Reaction Kit (Applied Biosystems, Foster City, CA, USA), and then analysed with an ABI PRISM 3100 Genetic Analyzer (Applied Biosystems) in accordance with the manufacturer's instructions.

## Results

**Figure 1:**
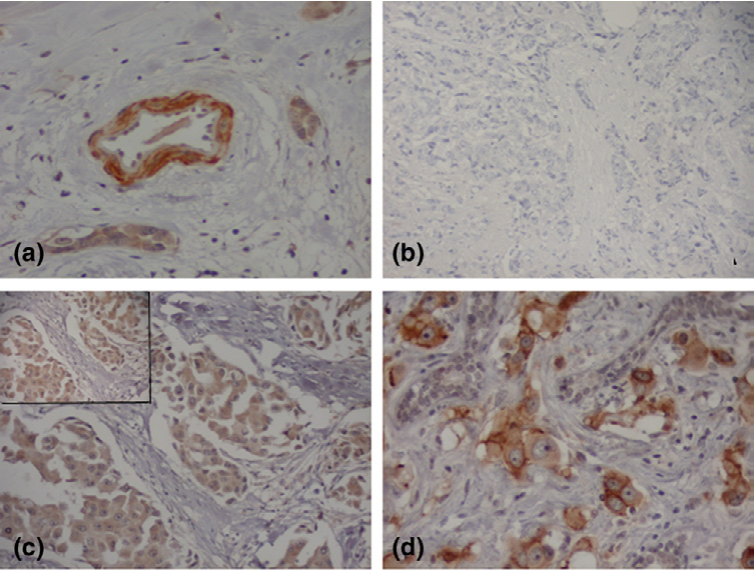
PDGFR-α expression in invasive breast carcinomas by immunohistochemistry (streptavidin-biotin-peroxidase). **(a) **Platelet-derived growth factor receptor α (PDGFR-α) expression in pericytes and smooth muscle cells of a blood vessel: internal control (original magnification × 200); **(b) **Absence of PDGFR-α expression in neoplastic cells (original magnification × 200); **(c) **PDGFR-α diffuse cytoplasmic expression in neoplastic cells (original magnification × 200; inset × 400); **(d) **Neoplastic cells showing strong and diffuse cytoplasmic PDGFR-α expression (original magnification × 400).

**Figure 2:**
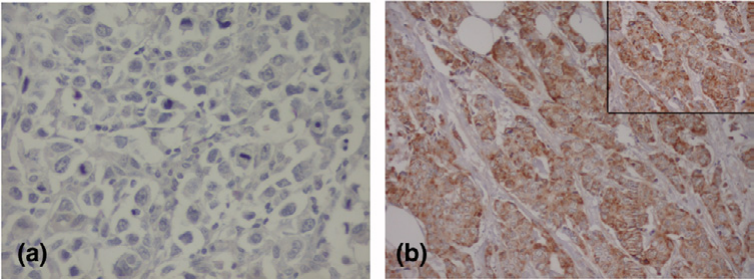
PDGF-A expression pattern studied by immunohistochemistry in invasive breast carcinomas. **(a) **Carcinoma without platelet-derived growth factor A (PDGF-A) expression (original magnification × 400); **(b) **Carcinoma with strong PDGF-A expression (original magnification × 200; inset × 400).

Immunohistochemistry for PDGFR-α was performed in 181 invasive ductal breast carcinomas and in the two breast cancer cell lines. PDGF-A immunostaining was performed in only 48 cases because of the unavailability of biological material. PDGFR-α cytoplasmic expression (Fig. 1) was found in 71 of 181 cases (39.2%), and PDGF-A expression (Fig. 2) was found in 27 of 48 cases (56.25%) (Table 2). Co-expression of PDGFR-α and PDGF-A was found in 10 cases and a significant correlation was observed between the expression of the receptor and the ligand (*P *= 0.0194). A positive correlation was also found between PDGFR-α expression and axillary lymph node status (*P *= 0.0079), Bcl2 expression (*P *= 0.0121) and HER-2 expression (*P *= 0.0265) (Table 3). Both cell lines showed an absence of PDGFR-α expression, but MCF-7 showed a low expression of PDGF-A, whereas HS578T did not express it at all.

**Figure 3:**
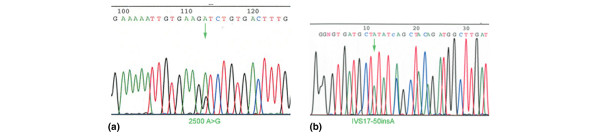
Platelet-derived growth factor receptor α (PDGFR-α) sequencing results for exon 18 (forward strand). **(a) **2500A→G (1834V) DNA sequence alteration; **(b) **IVS17-50insA intronic insertion.

Five of the six cases sequenced for exon 12 did not show any nucleotide sequence alteration and were considered normal. PCR-SSCP followed by direct sequencing of exons 12 and 18 of *PDGFR-α *revealed some alterations (Fig. 3): 2500A→G and 2529T→A in exon 18 (in the same case) and a further two in distinct cases: 2472C→T (exon 18) and 1701G→A (exon 12). The intronic insertion IVS17-50insA in exon 18 was found in all cases sequenced, although four cases were heterozygous and the other two homozygous for the inserted A (Table 4). The two cell lines studied also presented the homozygous intronic insertion IVS17-50insA, but no other DNA sequence alterations were found among them.

The expression of PDGFR-α varied between the cases with the alterations described above and did not show any correlation with the nucleotide alterations found.

## Discussion

PDGFR is a growth factor receptor with intrinsic tyrosine kinase activity and is deregulated in several human diseases. Breast carcinomas are known to express PDGF; however, there have been few studies on PDGF receptors in breast neoplasias, most of them related to the β subunit [10].

In our study, 71 (39.2%) of the 181 invasive carcinomas analysed expressed PDGFR-α. Slightly different results were obtained by Leu and colleagues [11]: using a high-density tissue microarray (TMA), they analysed PDGFRs expression in several human malignancies and found PDGFR-α expression in 98% of the 49 breast carcinomas studied. This high positivity could be justified by the use of a different antibody or by a distinct evaluation, although there is no description of the methodology used in that study. In the same study, Leu and colleagues [11] demonstrated PDGFR-α overexpression in other types of carcinoma, such as ovary, prostate, colon and lung. A high level of expression was also observed in ovarian carcinomas (81%) in comparison with the study by Matei and colleagues [12], which obtained 68.3% of PDGFR-α expression. These data suggest that the study of Leu and colleagues [11] overestimated values of PDGFR-α expression in different tumour types.

Because the deregulation of PDGFR signalling can lead to an autocrine or a paracrine stimulation of the tumour cells, we also evaluated the expression of PDGF-A, which is the major ligand of this receptor.

Twenty-seven (56.25%) of 48 carcinomas analysed showed expression of PDGF-A, and we found a statistically significant correlation with PDGFR-α, suggesting a mechanism of autocrine stimulation. This autocrine expression might have a causal role in the development of a variety of human cancers and, for example, seems to be involved in the development of high-grade sarcomas and gliomas [13,14]. De Jong and colleagues [15] reported co-expression of PDGF-A/PDGFR-α in epithelium, stroma and endothelium of invasive breast carcinomas and obtained indications about possible autocrine and paracrine mechanisms in the stroma, where they might be responsible for a baseline stromal proliferation, and in the endothelium, where they promote a basic level of angiogenesis. The simple presence of ligand/receptor combinations does not necessarily indicate this type of mechanism, especially when the ligand and the receptor are produced in spatial sites distant from each other [15]. In this context, an elegant way to demonstrate the PDGFR-α activation is to assay phosphorylated PDGFR-α (p-PDGFR-α) by immunohistochemistry. However, until now p-PDGFR-α antibodies have been reliable only in frozen samples that were not available in our series.

To investigate the role of PDGFR-α in neoplastic proliferation and progression, we correlated PDGFR-α immunoexpression with prognostic factors and molecular markers previously studied in this series. We did not obtain significant differences related to the classic prognostic factors, such as tumour size, histological grade and oestrogen receptor status. However, we found an association between PDGFR-α expression and positive axillary lymph node status, suggesting that PDGFR-α-positive tumours have a more aggressive phenotype.

We also obtained a correlation between PDGFR-α expression and Bcl2 expression. Bcl2 is an anti-apoptotic protein overexpressed in about 60 to 80% of breast cancers [16], and several studies suggest that the low apoptotic response caused by that overexpression allows the accumulation of genetic alterations that might be important in breast cancer metastatic potential [17,18]. In our study, 66.7% of the carcinomas expressing PDGFR-α present Bcl2 co-expression, and we speculate that PDGFR-α might be activating anti-apoptotic routes such as the Bcl2 pathway.

The association found between PDGFR-α expression and HER-2 expression is quite interesting, and probably proves the previous results about the correlation of PDGFR-α pathway with aggressiveness. HER-2 amplification/overexpression occurs in 30% of human breast cancers and is associated with biological aggressiveness and shortened disease-free survival and overall patient survival [19,20]. Our results seem to indicate that there is a relationship between these two receptors: most carcinomas that expressed PDGFR-α also expressed HER-2, and the absence of PDGFR-α was also found at a higher frequency in carcinomas without HER-2 expression. PDGFR-α and HER-2 are both tyrosine kinase receptors and although they belong to different subfamilies they can give rise to similar cellular/biological effects [21]. The coexistence of these receptors might contribute to neoplastic proliferation but might also influence tumour cell survival.

These results demonstrate that PDGFR-α expression is correlated with certain aggressiveness parameters of invasive breast carcinomas, and corroborate other results [22,23] showing PDGFR-α overexpression associated with aggressive characteristics in ovarian and renal cell carcinomas, respectively.

As far as we know there have been no studies on PDGFR-α expression in breast cancer cell lines; here we report that none of the cell lines studied express PDGFR-α. Breast cancer cell lines are known to secrete PDGFs, and, as Bronzert and colleagues [24] reported, MCF-7 shows some expression of PDGF-A, as we demonstrated in our work. No information about PDGF-A status in the HS578T cell line is available and in our study it did not show any expression. Ligand production by breast cancer cell lines might therefore have a role in mediating paracrine stimulation of tumour growth, affecting other cells in the microenvironment [15].

PDGFR-α overexpression can occur by genetic amplification or through activating mutations. PDGFR-α amplification has already been investigated in some tumour types, such as oesophageal squamous cell carcinoma [25], pulmonary artery intimal sarcomas (where it was demonstrated that PDGFR-α amplification is strongly associated with the development of this type of neoplasia) [26] and glioblastoma, occurring in 8 to 16% of cases [14]. However, PDGFR-α amplification in breast carcinomas was detected only by Daigo and colleagues [27], with controversial results: by array-comparative genomic hybridization, 21% of the cases presented gene amplification, and by metaphase-comparative genomic hybridization no amplification was detected.

As regards PDGFR-α genetic alterations, most studies have been performed in GIST, demonstrating that activating mutations in exons 12 and 18 has a major role in the development of these type of tumours [6,7]. It has also been shown that this receptor can be a therapeutic target for a recently developed drug, imatinib mesylate.

Because PDGFR-α amplification has shown controversial results and does not seem to be correlated with a response to imatinib therapy, we decided to search for the activating mutations frequently observed in GISTs in our sample of breast carcinomas, as well as in the two breast cancer cell lines MCF-7 and HS578T.

We found some alterations in PDGFR-α gene sequence: 2500A→G, 2529T→A and 2472C→T in the tyrosine kinase II domain, all in exon 18, and 1701G→A in the transmembrane domain of exon 12. However, none of these alterations corresponded to those described in GISTs [5-7] or even to the unique mutation found in breast phyllodes tumours [28]. We still observed an intronic insertion IVS17-50insA, in all cases sequenced for exon 18, including the two cell lines. This insertion has already been described [28] in a breast phyllodes tumour and is observed in the general population, suggesting that it might be a polymorphism. In other histological tumour types, PDGFR-α-activating mutations are unknown [29].

Although the DNA sequence alterations found do not have any effect on protein structure and/or function, it is necessary to emphasize that the alterations with amino acid change that were found in the tyrosine kinase domain could be important, because they can affect autophosphorylation (activation) and consequent cellular effect (signalling), allowing constitutive activation of the receptor. The cases with gene sequence alterations do not present clinical or anatomical-pathological features that distinguish them from the other cases, so the possible consequences of the alterations described should be evaluated by functional studies. Further studies are also necessary to define the role of PDGFR-α in breast oncogenesis, as well as the inherent molecular mechanisms of this process. It is important to stress that PDGFR-α and other protein tyrosine kinases can be activated by mechanisms different from gene mutations, such as gene fusion and amplification, autocrine and paracrine receptor stimulation by its ligand, loss of phosphatase activity, cross-activation by other kinases and promoter activation/inactivation via methylation/demethylation.

## Conclusion

Our results demonstrated the presence of PDGFR-α expression in 39.2% of invasive ductal carcinomas and that this expression was correlated with aggressiveness parameters, such as the presence of regional lymph node metastasis, HER-2 expression and Bcl2 expression, and also showed an association with PDGF-A ligand expression. Although the PDGFR-α mutations detected in this study were not correlated with protein expression, other mechanisms, such as gene amplification or constitutive activation of a signalling pathway, could explain the overexpression observed in our study and still can sustain PDGFR-α as a potential therapeutic target in breast cancer.

## Abbreviations

CGH = comparative genomic hybridization; GIST = gastrointestinal stromal tumour; PCR-SSCP = polymerase chain reaction-single-stranded conformational polymorphism; PDGF = platelet-derived growth factor; PDGFR-α and PDGFR-β = platelet-derived growth factor receptor α and β.

## Competing interests

The authors declare that they have no competing interests.

## Authors' contributions

IC performed immunohistochemistry, PCR-SSCP and statistical analysis, and analysed the data and drafted the article. FM contributed significantly to the analysis and interpretation of the immunohistochemistry data. AM was the responsible for the cell culture results. RR contributed to the mutation analysis. FS coordinated the study and took a role in the supervision and final approval of the article. All authors read and approved the final manuscript.

## Figures and Tables

**Table 1:** Primer sequence, annealing temperatures and fragment length, for PDGFR-α exons

Exon	Primer sequence (5'→3')	Annealing temperature (°C)	Fragment length (base pairs)
12	F, TCCAGTCACTGTGCTGCTTC; R, GCAAGGGAAAAGGGAGTCTT	58	260
18	F, ACCATGGATCAGCCAGTCTT; R, TGAAGGAGGATGAGCCTGACC	60	251

F, forward primer; PDGFR-α, platelet-derived growth factor receptor α; R, reverse primer.

**Table 2:** PDGFR-α and PDGF-A expression in invasive ductal breast carcinomas

Expression	PDGFR-α, *n *(%)	PDGF-A, *n *(%)
Negative	110 (60.8)	21 (43.75)
Positive	71 (39.2)	27 (56.25)
Total	181 (100)	48 (100)

PDGFR-α, platelet-derived growth factor receptor α; PDGF-A, platelet-derived growth factor A.

**Table 3:** Correlation between PDGFR-α expression, PDGF-A expression and clinical–pathological parameters in invasive ductal carcinomas

Parameter	PDGFR-α negative	PDGFR-α positive	*P*
PDGF-A			
Negative	19 (52.7%)	2 (16.7%)	
Positive	17 (47.3%)	10 (83.3%)	0.0290
Age (*n*)	56.315 ± 11.913 (108)	52.986 ± 12.811 (70)	NS (0.0788)
Tumour size, mm (*n*)	31.406 ± 22.056 (106)	30.101 ± 22.655 (69)	NS (0.7057)
Histological grade			
I	20 (19.4%)	9 (12.8%)	
II	38 (36.9%)	34 (48.6%)	
III	45 (43.7%)	27 (38.6%)	NS (0.2595)
Axillary lymph node status			
Negative	47 (48%)	18 (27.3%)	
Positive	51 (52%)	48 (72.7%)	0.0079
Oestrogen receptor status			
Negative	30 (27.8%)	19 (26.7%)	
Positive	78 (72.2%)	52 (73.3%)	NS (0.8813)
p53			
Negative	38 (54.3%)	21 (53.8%)	
Positive	32 (45.7%)	18 (46.2%)	NS (0.9648)
MIB-1 (*n*)	19.449 ± 17.484 (49)	12.333 ± 12.584 (15)	NS (0.1491)
HER-2			
Negative (0/1+)	54 (57.4%)	26 (40%)	
2+	17 (18.1%)	10 (15.4%)	
3+	23 (24.5%)	29 (44.6%)	0.0265
Bcl2			
Negative	29 (59.2%)	15 (33.3%)	
Positive	20 (40.8%)	30 (66.7%)	0.0121
Angiogenesis index (*n*)	43.393 ± 26.498 (55)	43.633 ± 27.635 (18)	NS (0.9737)
Patient survival, months (*n*)	24.917 ± 19.500 (48)	21.067 ± 20.405 (15)	NS (0.5115)

NS, not statistically significant; PDGFR-α, platelet-derived growth factor receptor α;

PDGF-A, platelet-derived growth factor A.

**Table 4:** Results of PDGFR-α gene mutations screening in invasive breast carcinomas

*PDGFR-α *gene sequence alterations	Exon 12	Exon 18
Normal	12/13	7/13
1701G→A (P567P)	1/13	-
2500A→G (I834V)	-	1/13
2529T→A (I843N)	-	1/13
2472C→T (V824V)	-	1/13
IVS17-50insA	-	6/13

PDGFR-α, platelet-derived growth factor receptor α.
